# High-flow nasal cannula use in pediatric patients for other indications than acute bronchiolitis—a scoping review of randomized controlled trials

**DOI:** 10.1007/s00431-023-05234-3

**Published:** 2023-11-14

**Authors:** Ilari Kuitunen, Heli Salmi, Elina Wärnhjelm, Saija Näse-Ståhlhammar, Panu Kiviranta

**Affiliations:** 1 Institute of Clinical Medicine and Department of Pediatrics, University of Eastern Finland, Puijonlaaksontie 2, 70210 Kuopio, Finland; 2 Department of Pediatrics, Kuopio University Hospital, Kuopio, Finland; 3 Department of Pediatrics, Helsinki Childrens Hospital, Helsinki, Finland; 4 Department of Anesthesiology, Helsinki Childrens Hospital, Helsinki, Finland; 5 Finnish Medical Society Duodecim, Helsinki, Finland

**Keywords:** High-flow nasal cannula, **A**cute bronchiolitis, Neonatal care, Pediatric patients

## Abstract

The objective of the study is to summarize current literature on high-flow nasal cannula (HFNC) use for different indications in pediatric patient excluding acute bronchiolitis and neonatal care. The study design is a systematic scoping review. Pubmed, Scopus, and Web of Science databases were searched in February, 2023. All abstracts and full texts were screened by two independent reviewers. Randomized controlled trials focusing on HFNC use in pediatric patients (age < 18 years) were included. Studies focusing on acute bronchiolitis and neonatal respiratory conditions were excluded. Study quality was assessed by Cochrane risk of bias 2.0 tool. The main outcomes are patient groups and indications, key outcomes, and risk of bias. After screening 1276 abstracts, we included 22 full reports. Risk of bias was low in 11 and high in 5 studies. We identified three patient groups where HFNC has been studied: first, children requiring primary respiratory support for acute respiratory failure; second, perioperative use for either intraprocedural oxygenation or postoperative respiratory support; and third, post-extubation care in pediatric intensive care for other than postoperative patients. Clinical and laboratory parameters were assessed as key outcomes. None of the studies analyzed cost-effectiveness.

*Conclusion*: This systematic scoping review provides an overview of current evidence for HFNC use in pediatric patients. Future studies should aim for better quality and include economic evaluation with cost-effectiveness analysis.

*Protocol registration*: Protocol has been published https://osf.io/a3y46/.

**What is Known:**

*• High flow nasal cannula has been effective in acute bronchiolitis and neonatal respiratory care.*

*• The use of HFNC on other conditions is also common and increasing, but the evidence supporting this has not been previously summarized.*

**What is New:**

*• We found that HFNC has been studies in relatively few studies in children for other indication than bronchiolitis.*

*• We indetified three main patient populations for which HFNC has been studied: perioperative patients, postintubation patients in intensive care units, and as primary support in acute respiratory failures. None of the studies have estimated possible cost-effectiveness of HFNC, compared to alternative strategies.*

## Introduction

High-flow nasal cannula (HFNC) therapy has rapidly gained popularity as respiratory support. HFNC therapy has been proved effective in various indications in neonatal care and acute bronchiolitis in infants [1–3]. In acute bronchiolitis the HFNC has reduced treatment failure rate compared to conventional oxygen treatment (COT), but it has had similar effectiveness as continuous positive airway pressure (CPAP) [4, 5]. In adults, previous systematic reviews have found HFNC beneficial in preventing escalation to intubation in acute hypoxemic respiratory failure, in preventing extubation failure, and in improving procedural oxygenation [6–9]. Because of the favoring evidence in these patient groups, the use of HFNC has expanded beyond neonatal respiratory support and bronchiolitis treatment in pediatrics. Simultaneously, there is ongoing effort to reduce the overuse of HFNC in acute bronchiolitis [10, 11]. There are no previous systematic summaries about HFNC use as primary respiratory support for other indications in the pediatric population. A recent systematic review found that HFNC use was associated to higher likelihood of extubation failure in young children [12]. Expanding HFNC use to new patient groups without evidence could also have negative effects such as increased costs and length of hospitalization, prolonged exposure to supplementary oxygen, and delayed escalation of respiratory support.

Previous randomized studies in children have typically compared HFNC to conventional oxygen therapy (COT), and continuous positive airway pressure (CPAP) [13]. The main hypothesis has been that HFNC would be more effective and provide benefits over COT, but be non-inferior to CPAP and better tolerated [14, 15]. Intervention tolerability is especially important in younger children.

To provide better knowledge on current evidence and to guide future studies, we aim to systematically evaluate for which indications HFNC has been studied in randomized controlled trials in pediatric patients.

## Methods

### Study design and search process

We conducted a systematic scoping review. We searched Pubmed, Scopus, and Web of Science databases in February, 2023. The complete search strategy is provided in Supplementary Materials. Two authors independently screened each abstract and full texts. Cases with conflicting decisions were decided either by mutual consensus or third-party opinion. All authors participated in the screening process.

We have reported our scoping review according to the Scoping review extension for Preferred Reporting Items in Systematic Reviews and Meta-analyses (PRISMA-ScR) [16].

### Inclusion and exclusion criteria

We used following PICOS (patients, interventions, comparator, outcome, and study design) as our inclusion criteria. Patients had to be pediatric patients, and we defined pediatric patients as children younger than 18 years. Intervention was high-flow nasal cannula therapy. HFNC was defined by the authors in the included studies. Control intervention or comparator could either be standard low flow oxygen therapy or noninvasive continuous positive airway pressure therapy or other support mode (for example, laryngeal mask airway). We did not specify any pre-selected outcome as either inclusion or exclusion criteria. Study design had to be parallel group randomized controlled trial. Crossover, quasi-experimental, or cluster randomized trials were excluded.

We decided to exclude studies only focusing on acute bronchiolitis in infants, as the evidence regarding this indication is rather solid and covered already by several systematic reviews. Similarly, we decided to exclude all studies which focused on respiratory care of preterm infants and full-term newborns during transition to extrauterine life. However, we included studies where high-flow nasal cannula was used in postoperative care as post-extubation therapy (for example, cardiothoracic surgery for congenital cardiac defects). We excluded studies that did not present original results. Furthermore, we excluded non-English literature.

### Main outcomes

Our main outcome for this scoping review was to identify the current indications for which the HFNC has been studied in randomized settings [17]. As we aimed especially to analyze the potential effectiveness of the intervention, we decided to focus on parallel group randomized controlled trials. These are typically the highest standard for evidence of effectiveness. Furthermore, we aimed to analyze the control interventions and the specific design of randomized studies (non-inferiority, superiority, etc.). Finally, we aimed to analyze the most used outcomes. We expect that main outcomes can be stratified in to three themes: clinical outcomes, laboratory parameter outcomes, and cost-effectiveness outcomes.

### Critical appraisal

We assessed the risk of bias in the each of the included study by using Cochrane risk of bias 2.0 tool [18]. As the tool is designed to be outcome specific, we decided to conduct the assessment based on the intended primary outcome. Risk of bias analysis was performed by one author with prior expertise of this method (I.K.). Risk of bias figures were generated by using the Robvis shinyapp [19].

### Data extraction

Data was extracted by one author and validated by a second author to reduce potential extraction errors. For this scoping review we extracted the following information: authors, journal, title, publication year, study period, country, study setting, intervention, control interventions, inclusion criteria, exclusion criteria, main outcomes, and secondary outcomes.

### Permissions and ethics

Permissions for publication were not needed due to study designs. Similarly, our study did not need ethical committee evaluation.

### Protocol registration

This review protocol was registered in Open Science Framework (https://osf.io/a3y46/).

## Results

### Search results and study characteristics

**Fig. 1:**
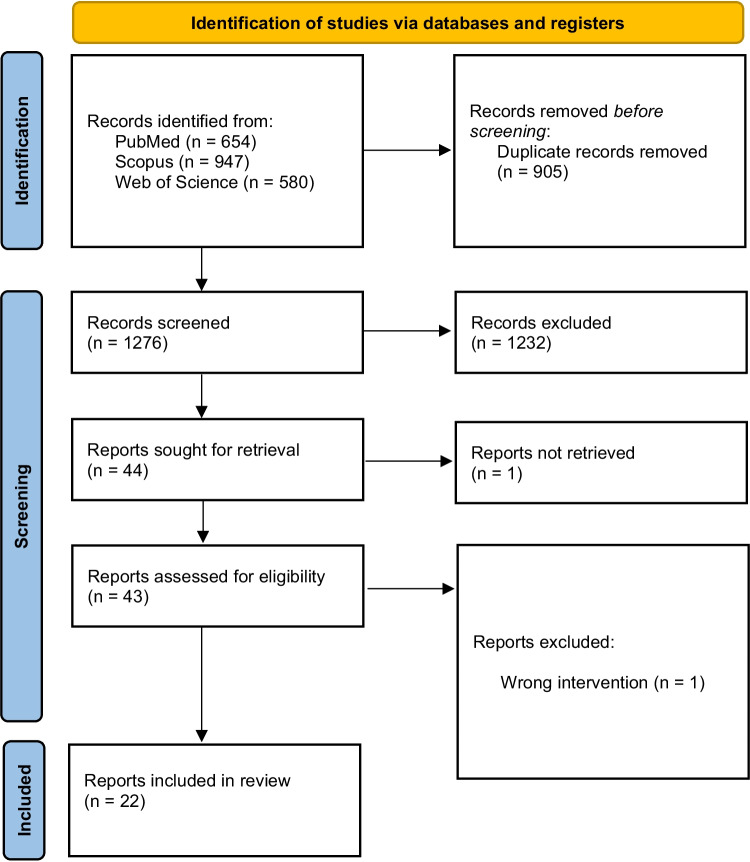
PRISMA flow chart of the study selection process

We screened 1276 abstracts and further assessed 43 full reports. Finally, 22 studies were included [20–41] (Fig. 1). Of these, 10 were conducted in Europe, 8 in Asia, 2 in Australia, 1 in South America, and 1 in Africa (Table 1). All studies were conducted in the 2010s or 2020s. Three of the included studies were single blinded and 19 were unblinded.

**Table 1:** Characteristics of the included studies grouped by study setting

Study	Country	Study period	Desing	Blinding	Setting	Patients	Intervention	Control 1	Control 2	Primary outcomes
**Emergency departments or general wards**										
Ballestero et al. (2018)	Spain	2012–2015	Pilot trial	Non-blinded	ED	Moderate-to-severe asthma exacerbations	HFNC	COT	N/A	Change in asthma severity
Benitez et al. (2019)	Paraguay	2017	Superiority trial	Non-blinded	ED	Severe and moderate asthmatic crises	HFNC	COT	N/A	Change in asthma severity
Franklin et al. (2021)	Australia	2016–2017	Feasibility trial	Non-blinded	ED and GW	Acute hypoxemic respiratory failure	HFNC	COT	N/A	Proportion of children when treatment failure of the allocated oxygen therapy occurred
Franklin et al. (2023)	Australia and New Zealand	2017–2020	Superiority trial	Non-blinded	ED	Acute hypoxemic respiratory failure without bronchiolitis	HFNC	COT	N/A	Length of hospital stay
Maitland et al. (2021)	Kenya and Uganda	2017–2020	Superiority trial	Non-blinded	ED and GW	Severe WHO pneumonia with hypoxemia	HFNC	COT	Permissive hypoxemia	Mortality at 48-h post-randomization and deaths to day 28
Sitthikarnkha et al. (2018)	Thailand	2014–2015	Superiority trial	Non-blinded	ED	Acute respiratory distress	HFNC	COT	N/A	Failure of treatment
**Operative patients**										
Roncin et al. (2020)	France	2018–2019	Pilot trial	Single-blinded	MRI	Patients needing MRI	HFNC	COT	N/A	The ratio of the atelectasis volume to the total pulmonary volume in MRI
Duan et al. (2021)	China	2018	Superiority trial	Non-blinded	OP	Patients scheduled for percutaneous closure of a heart defect	HFNC	COT	N/A	Lowest oxygen saturation (SpO2)
Klotz et al. (2020)	Germany	2016–2017	Pilot trial	Non-blinded	OP	Gastrointestinal tract endoscopy patients	HFNC	COT	N/A	The number of events of respiratory instability defined by prespecified criteria (hypoxia, hypercapnia, apnea)
Kumar et al. (2022)	India	2018–2019	Superiority trial	Non-blinded	OP	Elective cardiac surgery for acyanotic congenital cardiac defects under cardiopulmonary bypass	HFNC	CPAP	N/A	Arterial partial pressure of CO2 (pCO2) compared with NIV
Lee et al. (2021)	South-Korea	2019	Superiority trial	Non-blinded	OP	Children receiving general anesthesia for operation for 2 h or more	HFNC	COT	N/A	Lung ultrasound score
Ran et al. (2022)	China	2020–2021	Non-inferiority trial	Single-blinded	OP	Ambulatory, oral surgery patients	HFNC	LMA	N/A	CO2 accumulation
Riva et al. (2018)	Switzerland	N/A	Superiority trial	Single-blinded	OP	Elective operation patients,	HFNC 100% FiO2	HFNC 30% FiO2	COT	Apnea time until one of the termination criteria was reached
Sharluyan et al. (2021)	Spain	2015–2019	Superiority trial	Non-blinded	OP	Bronchoscopy patients	HFNC	COT	N/A	The proportion of patients experiencing oxygen desaturation during the procedure
**Pediatric intensive care units**										
Akyildiz et al. (2018)	Turkey	2014–2016	Superiority trial	Non-blinded	PICU	All cause PICU patients after extubation	HFNC	COT	N/A	The changes of respiratory, hemodynamic, and radiologic parameters
Chisti et al. (2015)	Bangladesh	2011–2013	Superiority trial	Non-blinded	PICU	Severe WHO pneumonia with hypoxemia	HFNC	CPAP	COT	Treatment failure (i.e., clinical failure, intubation and mechanical ventilation, death)
Enayati et al. (2021)	Iran	2020	Superiority trial	Non-blinded	PICU	Cardiac surgery patients	HFNC	COT	N/A	Atelectasis and PaO2/FiO2 ratio
Liu et al. (2020)	China	2018–2019	Equivalence trial	Non-blinded	PICU	Acute mild to moderate respiratory failure due to pneumonia	HFNC	CPAP	N/A	Treatment failures and intubations, duration of hospital stay and PICU stay, mortality
Ramnarayan et al. (2018)	UK	2015–2016	Pilot trial	Non-blinded	PICU	All cause PICU patients both before and after extubation	HFNC	CPAP	N/A	Feasibility outcomes for future RCT
Ramnarayan et al. (2022a)	UK	2019–2020	Non-inferiority trial	Non-blinded	PICU	All cause PICU patients after extubation	HFNC	CPAP	N/A	Time to liberate from respiratory support, mortality, reintubation rate, hospital stay duration
Ramnarayan et al. (2022b)	UK	2019–2020	Non-inferiority trial	Non-blinded	PICU	PICU patients requiring noninvasive respiratory support	HFNC	CPAP	N/A	Time to liberate from respiratory support, mortality, reintubation rate, hospital stay duration
Testa et al. (2014)	Italy	2012–2013	Superiority trial	Non-blinded	PICU	Pediatric cardiac surgical patients	HFNC	COT	N/A	Comparison of arterial PaCO2 post-extubation

*COT *conventional oxygen therapy, *ED *emergency department, *GW *general ward, *HFNC *high-flow nasal cannula, *LMA *laryngeal mask airway, *MRI *magnetic resonance imaging, *PICU *pediatric intensive care unit, *OP *operative patients

### Risk of bias

**Fig. 2:**
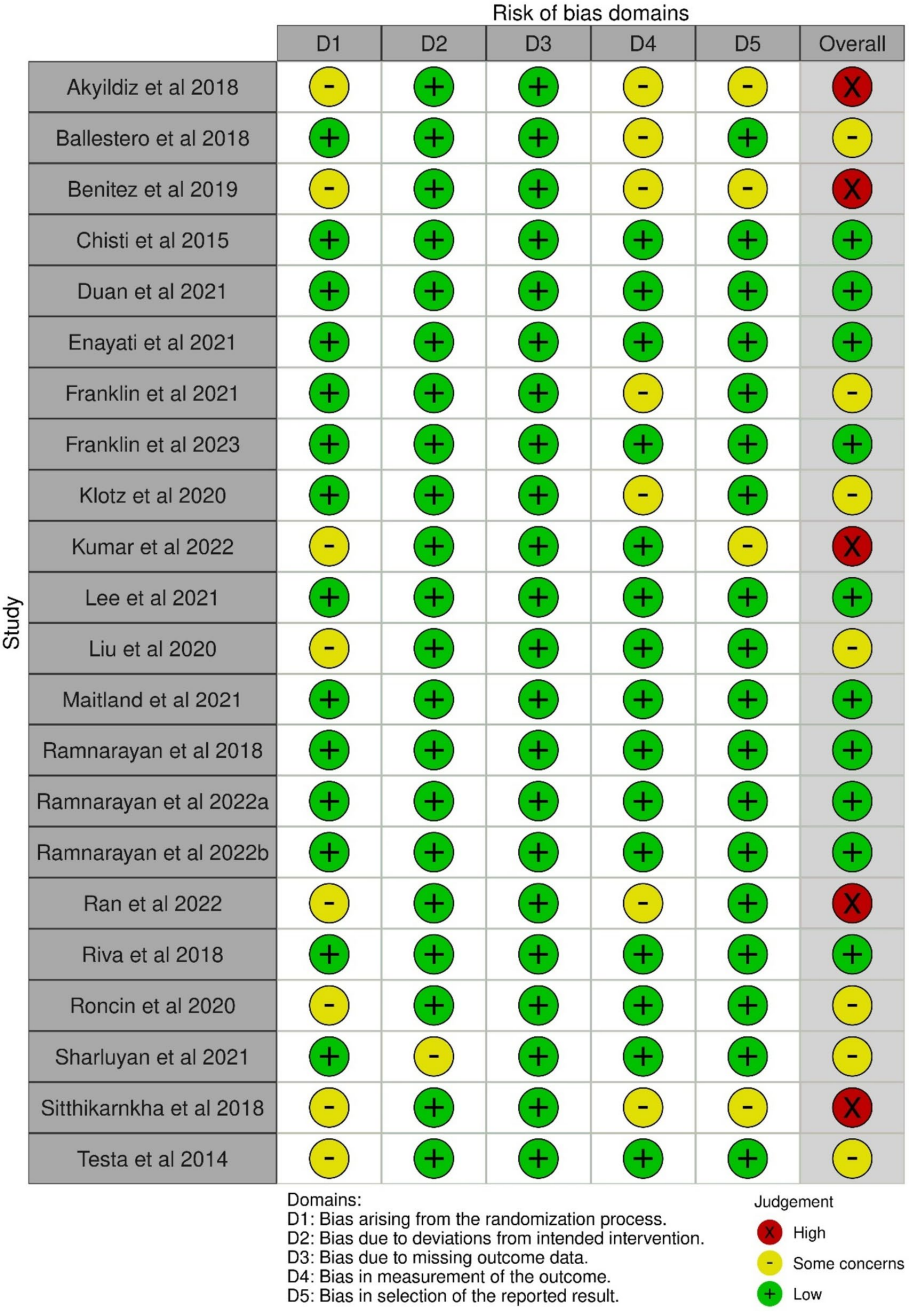
Risk of bias assessed in five domains, and overall by using Cochrane risk of bias 2.0 tool

Overall risk of bias was low in 11 studies, had some concerns in 7 studies, and was high in 5 studies (Fig. 2). Most issues were due to bias in randomization process and in outcome measurement. The majority of studies were completely unblinded and caused some problems in the outcome assessment. Furthermore, some issues were seen in reporting the outcomes, as not all studies had prespecified protocol presented or referenced.

### Indications and control interventions

The indications and patient groups had high variability. Eight studies were conducted in PICU patients, five studies focused on emergency departments, and eight studies were conducted in perioperative care patients. The patients could be categorized into three main groups: first, patients requiring primary respiratory support for acute respiratory failure; second, patients in the perioperative period needing respiratory support during or after the procedure; third, PICU patients with HFNC as post-extubation respiratory support for other than perioperative use. The control intervention was conventional oxygen therapy in 15 studies, CPAP in six studies, and laryngeal mask airway in one study (Table 1).

### Study design

All included studies were randomized controlled trials, of which three were single blinded. The specific designs were superiority trial (13 studies), pilot or feasibility trial (5 studies), non-inferiority trial (3 studies), and equivalence trial (1 study) (Table 1).

### Most frequently reported outcomes

Most frequently reported outcomes were clinical outcomes, such as asthma severity, reintubation rate, mortality, and length of stay (PICU and overall). Laboratory outcomes were used especially in perioperative studies where the main interest was gas exchange, typically assessed by arterial pCO2, pO2, and pO2 to FiO2 ratio. Few studies assessed imaging findings, such as presence of atelectasis by lung ultrasound or magnetic resonance imaging. None of the included studies provided cost-effectiveness analysis (Table 1). Adverse effects were infrequently and incompetently reported (presence of hyperoxia or cumulative exposure to supplementary oxygen, rate of accumulation of air into intestines with effect on incidence of nausea and vomiting or on the time needed to achieve full enteral feeds).

### Summary of reported results

Six studies analyzed HFNC utilization in emergency departments and general pediatric wards. Three of the studies indicated possible benefits associated with HFNC while the remaining three studies did not identify any significant difference between the HFNC and comparator interventions (Table 2). Additionally, eight studies examined the use of HFNC during procedures or in operated patients. Among these, four studies reported benefits (reduced atelectasis, improved oxygenation), and four of the studies reported no evidence of a benefit of HFNC use. Notably, none of the studies reported increased rates of adverse events (Table 2). Furthermore, eight studies analyzed HFNC use in the context of PICUs. Out these, five studies reported positive outcomes and concluded that HFNC is a feasible or non-inferior option to CPAP or superior to COT. Meanwhile, two studies did not detect differences between treatment groups, and one study found HFNC to be less effective than CPAP as post-extubation therapy (Table 2).

**Table 2:** Main results and author conclusions of the included studies

Study	Sample size	Author conclusion
**Emergency departments or general wards**		
Ballestero et al. (2018)	62	HFNC appears to be superior to conventional oxygen therapy for reducing respiratory distress within the first 2 h of treatment in children with moderate-to-severe asthma exacerbation
Benitez et al. (2019)	64	HFNC in the treatment of asthmatic crises did not show clinical benefits nor did reduce the stay time
Franklin et al. (2021)	563	HFNC outside ICU appears to be feasible in children with acute respiratory failure and the required proportion of escalation was lower compared to standard-oxygen
Franklin et al. (2023)	1567	HFNC used as the initial primary therapy in children aged 1 to 4 years with acute hypoxemic respiratory failure did not significantly reduce the length of hospital stay compared with standard oxygen therapy
Maitland et al. (2021)	1842	Respiratory support with HFNC showing potential benefit in mortality should prompt further trials
Sitthikarnkha et al. (2018)	98	HFNC therapy revealed a potential clinical advantage in respiratory distress compared with conventional respiratory therapy. The early use of HFNC in children with moderate‐to‐severe respiratory distress may prevent endotracheal tube intubation
**Operative patients**		
Roncin et al. (2020)	39	HFNC was associated with a lower atelectasis lung ratio compared to using a face bag-mask during anesthesia for children maintained with spontaneous ventilation
Duan et al. (2021)	200	HFNC could reduce the incidence of desaturation, the need for airway assisted ventilation and risk of carbon dioxide retention without causing hemodynamic instability or gastric distention. It is effective for pediatric patients with non-cyanotic congenital heart disease who require procedural sedation
Klotz et al. (2020)	50	HFNC did not increase respiratory stability in sedated children undergoing upper gastrointestinal tract endoscopy compared to COT
Kumar et al. (2022)	127	HFNC did not show improved CO2 washout over NIV; however, it did provide better oxygenation as measured by pO2 in arterial blood and pO2/FiO2 ratio immediate postextubation. Duration of mechanical ventilation and ICU stay were not affected by the choice of device
Lee et al. (2021)	98	Preventive use of HFNC after surgery improves the lung ultrasound score and reduces postoperative atelectasis compared to conventional oxygen therapy in infants and small children
Ran et al. (2022)	120	HFNO was not inferior to LMA for maintaining oxygenation and ventilation in patients undergoing pediatric ambulatory oral surgery under deep sedation under strict isolation from the oral cavity to the upper airway
Riva et al. (2018)	60	HFNC administered via nasal cannulas did not extend the safe apnea time for children weighing 10–20 kg compared with COT
Sharluyan et al. (2021)	104	HFNC offers optimized oxygenation during elective bronchoscopy with a significant reduction in desaturations and can be considered for oxygen administration
**Pediatric intensive care units**		
Akyildiz et al. (2018)	100	HFNC is better than COT, especially for the restoration of the respiratory and radiologic parameters. HFNC may have more advantages to reduce the risk of extubation failure in critically ill children
Chisti et al. (2015)	255	No difference in treatment failure was noted between patients in the bubble CPAP and HFNC groups
Enayati et al. (2021)	92	HFNC could improve the respiratory parameters and reduce postoperative pulmonary complications in infants following a congenital heart surgery
Liu et al. (2020)	84	HFNC is an effective and safe initial respiratory support treatment in children < 2 years with mild to moderate respiratory failure due to pneumonia, and the incidence of intubation and death is very low; concurrently, the comfort and tolerance of HFNC are better. To some extent, HFNC is a well-tolerated alternative to CPAP
Ramnarayan et al. (2018)	113	It is feasible to conduct a large national RCT of non-invasive respiratory support in the pediatric critical care setting in both step-up and step-down patients
Ramnarayan et al. (2022a)	533	Among critically ill children requiring noninvasive respiratory support following extubation, HFNC compared with CPAP following extubation failed to meet the criterion for noninferiority for time to liberation from respiratory support
Ramnarayan et al. (2022b)	573	Among acutely ill children clinically assessed to require noninvasive respiratory support in a pediatric critical care unit, HFNC compared with CPAP met the criterion for noninferiority for time to liberation from respiratory support
Testa et al. (2014)	89	HFNC had no impact on PaCO2 values. The use of HFNC appeared to be safe and improved PaO2 in pediatric cardiac surgical patients

## Discussion

In this systematic scoping review, we found that HFNC has been studied in a variety of pediatric patients and conditions. We identified three key patient groups: acute respiratory failure, perioperative care, and PICU post-extubation respiratory support. Key outcomes assessed were clinical and laboratory outcomes. None of the studies assessed cost-effectiveness.

The most studied patient groups and indications were patients needing primary respiratory support due to acute respiratory failure, followed by perioperative care and PICU post-extubation therapy. The indications were similar for which previous studies in adults have shown benefit or equal effectiveness of HFNC treatment compared to COT or CPAP therapies [42–44].

The most frequently used control intervention was COT. All studies comparing HFNC to COT aimed at showing the superiority of HFNC treatment. The second most used control intervention was CPAP, for which either non-inferiority or equivalence designs were used. The design choices were rational, as HFNC should provide benefit over COT and be at least non-inferior to CPAP to be a justified respiratory support mode.

Main outcomes were mostly clinical or laboratory parameters. However, the lack of adverse effect reporting and the complete missing of cost-effectiveness estimations were unfortunate, as in general novel therapies should be safe and preferably cost-effective. Previous systematic review in neonatal patients concluded that there is currently no evidence of HFNC cost-effectiveness against nCPAP in preterm patients [45]. In adult patients HFNC has shown cost-effectiveness in intubation or reintubation prevention in ICU patients, and for chronic obstructive pulmonary disease patients in chronic respiratory failure [46, 47]. A recent systematic review found that HFNC and CPAP were better than COT in preventing extubation failures in infants and young children [12]. In their review CPAP seemed to be the best performing post-extubation support, although the studies were conducted in relatively heterogenous patients.

Enhanced clarity and precision in patient population definitions within future studies would significantly contribute to the interpretability of results. For instance, the inclusiveness of a wide age range (1–14 years) within the same trial investigating acute asthma exacerbations could potentially confound findings. Physiologically, the nature of acute asthma considerably varies between a 1-year-old and a teenager [29]. Moreover, PICU studies have included both all-cause patients or cardiac surgery patients. Notably, trials focused solely on cardiac surgery patients have demonstrated outcomes that hold greater applicability in clinical settings due to the more well-defined patient population. Considering the broad spectrum of patient categories within the PICU, it is evident that HFNC is not the universal solution to all cases.

We detected issues in the risk of bias assessment in the original studies. Most of the issues came from the randomization process and outcome measurement. These issues should be remarked in future trials where the researchers should focus on proper allocation concealment and randomization process and describe those in depth in the final report. Furthermore, an attempt to blind at least outcome assessors in some parts of the studies should be made to improve the reliability. A positive sign was that we did not detect issues with missing outcome data.

This is the largest effort to gather systematic assessment of current literature on HFNC use outside of neonatal respiratory care and acute bronchiolitis infants. We performed a rigorous systematic assessment according to a pre-specified protocol and we did not have any major protocol deviations. Our scoping review provides a basis for future studies and reviews on the use of HFNC.

Our main limitation is the lack of non-English literature, as most likely we have missed some RCTs published in other languages. Furthermore, only one author performed the risk of bias assessment, which can be seen as a limitation. Furthermore, we did not proceed to meta-analysis due to substantial variation in the studies and indications and instead conducted a scoping review of current knowledge and evidence.

## Conclusion

In conclusion we found that HFNC has been studied in a variety of settings and indications in children. We identified three key patient groups where HFNC was studied: acute respiratory failure, perioperative care, and post-extubation respiratory support in PICU patients. Key outcomes assessed were clinical outcomes, and none of the studies assessed cost-effectiveness. Further studies should aim to better study quality and assess cost-effectiveness alongside the clinical effectiveness and treatment-related harms or adverse events.

## Data Availability

All data generated during the review process available upon request from the corresponding author.
